# Exceptional antibacterial and cytotoxic potency of monodisperse greener AgNPs prepared under optimized pH and temperature

**DOI:** 10.1038/s41598-021-82555-z

**Published:** 2021-02-03

**Authors:** Muhammad Riaz, Vishal Mutreja, Shweta Sareen, Bashir Ahmad, Muhammad Faheem, Nafeesa Zahid, Ghassan Jabbour, Jeongwon Park

**Affiliations:** 1 School of Electrical Engineering and Computer Science, University of Ottawa, Ottawa, ON K1N 6N5 Canada; 2 Department of Biological Sciences, International Islamic University Islamabad, Islamabad, 44000 Pakistan; 3 Division Chemistry, University Institute of Sciences, Chandigarh University, Gharuan, Mohali, Punjab India; 4 Department of Chemistry, Panjab University, Sector-14, Chandigarh, India; 5 Department of Botany, Mirpur University of Science and Technology (MUST), Mirpur, 10250 Azad Kashmir Pakistan; 6 Department of Electrical and Biomedical Engineering, University of Nevada, Reno, NV 89557 USA

**Keywords:** Biochemistry, Nanoscience and technology

## Abstract

In the present work, silver nanoparticles were prepared by using the extract of *Camellia Sinensis.* The extract contains phytochemicals which are mainly polyphenols acting as the natural reducing and stabilizing agents leading to the formation of uniformly dispersed and stabilized silver nanoparticles. The synthesis of silver nanoparticles was significantly influenced by the impact of the pH, as well as temperature conditions. It was found that at pH 5 and 25 °C, nanoparticles of different morphologies (spherical, polygonal, capsule) and sizes were formed. However, with the increase in temperature from 25 °C to 65 °C but at the same pH, these particles started attaining the spherical shape of different sizes owing to an increase in the reduction rate. Furthermore, for the reaction of the mixture at 65 °C, an increase in pH from 5 to 11 led to an increase in the monodispersity of spherically shaped nanoparticles, attributed to the hydroxide ions facilitated reduction. The prepared nanoparticles were investigated for their antibacterial activity using Nathan’s Agar Well-Diffusion method. It was found that AgNPs prepared at pH 9 and 65 °C demonstrated strong antibacterial activity against gram-negative *Escherichia coli* in contrast to gram-positive *Staphylococcus aureus*. In reference to the cytotoxic potency, the prepared AgNPs showed clear cytotoxicity for HeLa cells and showcased a close relationship between activity and concentration as evidenced by the decrease in the percentage (100 to 30%) of metabolically active cells up to 25 µM–75 µM concentration of silver nanoparticles.

## Introduction

Over the last two decades, noble metallic nanostructures are being explored owing to their superior physicochemical, electronic, and optical properties in contrast to their corresponding bulk materials^1–3^. The excellent material properties of gold and silver nanoparticles such as high surface area, tunable surface plasmon resonance, and surface functionalization, have afforded them in a variety of applications such as catalysis, antimicrobial, medical imaging, and targeted drug delivery for therapeutic management^4–6^. Specifically, silver is clinically non-toxic in low doses. Moreover, due to its anti-microbial efficacy, silver has been incorporated in surgical prosthesis^7^, filters for water purification^8^ as well as in nano-formulations. Different procedures have been followed for synthesizing silver nanoparticles (AgNPs) varying from electrochemical reduction, thermal decomposition, microwave irradiation, laser ablation, and chemical reduction of silver ions^9^. These methods may steer better size distribution and showcase better reproducibility, however, the involvement of expensive and complicated techniques and toxic chemicals limit the biological applications of nanoparticles.

Contrastingly, the biogenic synthesis of nanoparticles has gained substantive attention as it can be performed without employing organic solvents/toxic chemicals. Moreover, it involves a rather simple, eco-friendly, cost-effective, convenient, and scalable procedure that could be followed at ambient reaction conditions as well. The biogenic synthesis of AgNPs has been reported using fungi, bacteria, algae, and diatoms^10,11^. Another greener approach for the synthesis of AgNPs is the use of phytochemicals viz., terpenoids, flavonoids, and other polyphenolic compounds derived from various parts (root, leaves, stem) of the plant as reducing agents^12^. Additionally, these species also act as a stabilizing agent and further impart superior biological properties by synergetic mechanisms because of their inherited nature. Jha et al*.*^13^ reported that leaf extract of Geranium containing terpenoids aided the biosynthesis of AgNPs. In another report^14^, eugenol present in the *Cinnamomum zeylanicum* extracts led to the reduction of silver nitrate to AgNPs. Begum et al*.*^15^ reported that extracts from *Ocimum basilicum* (sweet basil) contained flavonoids, eugenol, and polyphenols that facilitated the reduction of silver ions. Vidhu et al*.*^16^ synthesized anisotropic silver nanoparticles of size ∼12 nm using aqueous seed extract of *Macrotyloma uniflorum*. Balkar et al*.*^17^ reported the use of banana peel extract for the preparation of silver nanoparticles, however, a coalescence of nanoparticles into nanoclusters was observed when the reaction mixture of extract and silver nitrate was incubated for 3 h. Subsequently, an incubation period of 15 days resulted in the formation of micro-aggregates.

Yallappa et al*.*^18^ reported the formation of spherical shaped particles in the range of 15–20 nm using *Acacia farnesiana* (Sweet acacia) seed extract as a bioreductant for silver ions under microwave irradiation. The marginal increase in redox potential to the positive value indicated the depletion of bioreductant in the seed extract that ultimately led to the reduction of silver ions to AgNPs. It was supported by the observed decrease in pH due to the release of hydrogen ions signifying the oxidation of the bio-reductant. Krishnaraj et al*.*^19^ demonstrated the preparation of AgNPs in the range of 20–30 nm using leaf extract of *Acalypha indica*. Prasad et al*.*^20^ reported the synthesis of AgNPs with an average size of 57 nm owing to the reduction of the silver ions present in the solution of silver nitrate by using aqueous leaves extract of *Moringa oleifera*. Literature survey has shown that a variety of plant extracts, as well as discarded agricultural wastes have been used for the synthesis of AgNPs. However, fine-tuning of reaction parameters (pH and temperature) for monodisperse and small size nanoparticles is lacking. Therefore, in the current study, we have investigated the effect of reaction parameters (pH and temperature) on the morphology and size of AgNPs. Further keeping in view, the perspective of developing new greener processes and the goodness of green tea such as its antioxidant nature, water solubility, and biocompatibility, its extract has been used as a reducing agent for the preparation of AgNPs. Further to employ useful constituents specifically catechins present in green tea^21,22^, prepared nanoparticles have been used for the antimicrobial activity. Additionally, in vitro cytotoxicity was also investigated against human cervical epithelioid carcinoma cells (HeLa cells).

## Material and methods

### Materials

Silver nitrate (99.9%), Alamar Blue, Dulbecco’s modified Eagle medium (DMEM), phosphate-buffered saline (PBS), sodium hydroxide (98%), hydrochloric acid (37%), nutrient agar, and nutrient broth were purchased from Sigma Aldrich. Green tea leaves (Legends of China) were collected from the natural food pantry store Billing Bridge shopping mall in Ottawa, Canada. All experiments were performed using deionized (DI) water obtained from the Advanced Research Complex (ARC) of the University of Ottawa.

### Preparation of green tea extract and silver nitrate solution

Initially, green tea leaves (10 g) were taken in DI water (100 mL) and the mixture was boiled for 40 min. The suspension thus obtained was cooled, filtered through Whatman filter paper No. 1 (25 μm), and kept under refrigeration. An aqueous solution (1 mM) of silver nitrate was prepared by using an appropriate amount of AgNO_3_ and DI water and stored in an amber color bottle at 4 °C.

### Greener synthesis of AgNPs

The AgNPs were synthesized by slight modification in a procedure reported elsewhere^23^. For this, 10 mL of as-prepared extract of green tea leaves was poured slowly into the as-prepared solution of silver nitrate (100 mL) under stirring in a round bottom flask. Subsequently, the pH of the solution adjusted to 5, 7, 9, and 11 using HCl and NaOH, and for each pH, four different reactions were carried out at temperatures 25, 45, 65, and 85 °C. Eventually, the reaction mixture turned brown in color which suggested the formation of nanoparticles, however, to ensure the completion of the reaction, stirring was continued for 30 min.

### Characterization of AgNPs

The absorption spectra of the as-prepared colloidal solution of nanoparticles were obtained using a UV–Vis Carry 7000 spectrophotometer within a range of 190 to 800 nm. Dynamic Light Scattering (DLS) and zeta potential measurements were recorded using water as solvent and Zetasizer Nano S90 (Malvern) at varying pH and temperature. Average hydrodynamic diameters were determined by taking a mean of at least three measurements in series using DLS. TEM micrographs were recorded with an FEI Tecnai G2 Spirit Twin TEM instrument. For determining the average size, ImageJ software (version ImageJ 1.52v) provided by the US National Institute of Health (http://imagej.nih.gov/ij) was used. The average size was evaluated by counting a minimum of 80 particles. For recording X-ray diffraction (XRD) measurement, firstly nanoparticles were recovered from colloidal solution by centrifugation at 1000 rpm for 20 min followed by redispersing in DI water. Subsequently, the suspension was drop cast repeatedly on a glass sample holder followed by heating at 60 °C till a sufficient amount is obtained on a holder. Finally, the measurement was carried out on Rigaku Ultima IV X-ray diffractometer using Cu Kα radiations, and data were recorded in steps of 0.04° with a scan step time of 1 s in the 2θ range of 10°–84.9°. The crystallite size was also determined from this measurement using Scherrer’s formula^23^. FT-IR spectra of as-prepared nanoparticles were recorded using Agilent Cary600 and its ATR (Attenuated Total Reflection) accessory.

### Antibacterial assay

The Antimicrobial activity of as-prepared AgNPs was tested by using Nathan’s Agar Well-Diffusion method (NAWD)^24^ against gram-negative bacterial strains such as *E. coli* and gram-positive strain *S. aureus*. For this purpose, the culture media was prepared by adding 1.5 g of nutrient agar and 1.3 g of nutrient broth in 100 mL of DI water. After autoclaving the solution and all the Petri plates, the solution was poured into the plates for solidification. The tested bacteria cultures 100 µL were dispersed uniformly on nutrient agar plates using a sterile cotton ball. Subsequently, four wells, each with a diameter of 6 mm were prepared using a sterilized nickel-plated brass cork borer, and 100 μL of prepared (20 µM) AgNPs solution was poured into these wells. After 24 h of incubation at 37 °C, the Zone of Inhibition (ZOI) was gauged in mm using water and cephradine as a negative and positive control respectively. Each measurement was carried out in triplets.

### Cytotoxic evaluation of AgNPs in human cervix epithelioid carcinoma cells

Human cervical epithelioid carcinoma (HeLa) cells were cultured in Dulbecco's modified Eagle medium (DMEM). This medium is fed with 10% fetal bovine serum, 1% penicillin–streptomycin, and 1% l-glutamine. The cells were maintained under cell culture conditions (37 °C, 5% CO_2_, and in a humidified environment).

Cytotoxicity was evaluated through the Alamar Blue^25^ assay and LIVE/DEAD viability stain^25^ with flow cytometry (FC). For this, twenty-five thousand HeLa cells were transferred to 96-well plates^25^ and permitted to adhere to the plates overnight under cell culture conditions. The AgNPs, after sterilization via 0.2 µm syringe filtration, in final concentrations of 0, 25, 50, and 75 μM were added to triplicate wells for timepoints of 1, 3, and 5 days of incubation. Media was changed every 3 days and particles were re-added at appropriate concentrations to the fresh media. At the appropriate time point, 10% Alamar Blue was added to the wells and incubated under cell culture conditions for 3 h. An aliquot of each well was placed in a 96-well plate^25^ and fluorescence was read on a plate read^25^. Further, in order to determine the count of cells in the plate at different time points, a previously prepared linear standard curve was used.

At the appropriate time points, wells were washed thrice using Dulbecco’s phosphate-buffered saline (DPBS) after the Alamar Blue assay, and cells were trypsinized with 0.25% trypsin-EDTA^25^. After inactivation with DMEM without phenol red, centrifugation of cell suspension was carried out for 10 min at 1000 rpm and 4 °C. Afterward, the supernatant was rejected and the pellet was resuspended in DMEM without phenol, 0.2 µM calcein-AM, and 16 µM ethidium homodimer-1. This solution was incubated for 20 min under cell culture conditions before FC. After performing standard compensation, the cell solution was evaluated with the FC^26^ using 488 nm excitation (530/30 bandpass) for calcein-AM (live cells) and 530 nm fluorescence (610/20 bandpass) for ethidium homodimer-1 (dead cells).

### Statistical analysis

Data of Alamar Blue and LIVE/DEAD viability assay are represented as means ± standard deviation (SD) of triplicate measurements for the respective assays. Statistical analysis was performed in R software by a one-way analysis of variance followed by a Tukey post-hoc test. # represents p < 0.05 compared to the day 1 value of the same concentration. * represents p < 0.05 compared to different concentrations within the same time point.

## Results and discussion

### Characterization of prepared silver nanoparticles

UV–Visible spectra of prepared nanoparticles were recorded to determine the surface plasmon resonance (SPR), band. A qualitative idea regarding the shape and size distribution of nanoparticles can be obtained from the position, width, and shape of the SPR band^27^. For instance, a single narrow SPR band suggests the occurrence of spherical monodisperse particles whereas broadband indicates the wide distribution of particle sizes^23^. On the same line, in this study, the morphology and size of the nanoparticles and hence their plasmonic bands demonstrated variations upon changing reaction parameters such as pH and temperature.

**Figure 1:**
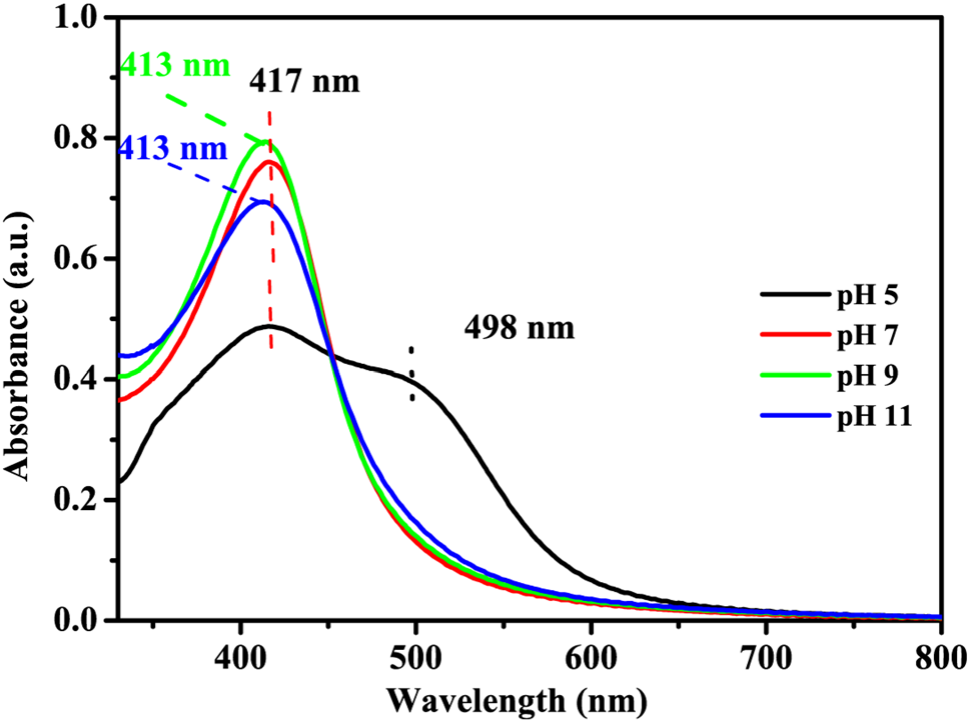
UV–Vis absorption spectra of silver nanoparticles prepared at different pH and temperature 25 °C.

In Fig. 1, at pH 5, a broad peak can be seen at 417 nm, which is a characteristic band for the AgNPs^28^. This broad SPR peak is followed by a shoulder at 498 nm indicating the existence of irregularly shaped nanoparticles in the solution^23,29^. However, this shoulder disappeared when the pH was increased to 7. As the pH was further increased to 9, the SPR peak at 417 nm became somewhat sharper and slightly shifted to 413 nm, which possibly indicated the formation of small-sized AgNPs. These observations signified the efficient reduction of silver ions in the presence of hydroxide ions (OH^−^) due to increased pH. Similar kinds of findings are also reported elsewhere^30,31^. Moreover, at higher pH conditions, all absorbance spectra depicted a single bell-shaped peak illustrating the formation of regular spherically shaped AgNPs^32^ which was further confirmed by TEM studies (vide infra). It is thus inferred that the increase in pH during reaction probably leads to the formation of more regular, spherically shaped, and small-sized AgNPs.

**Figure 2:**
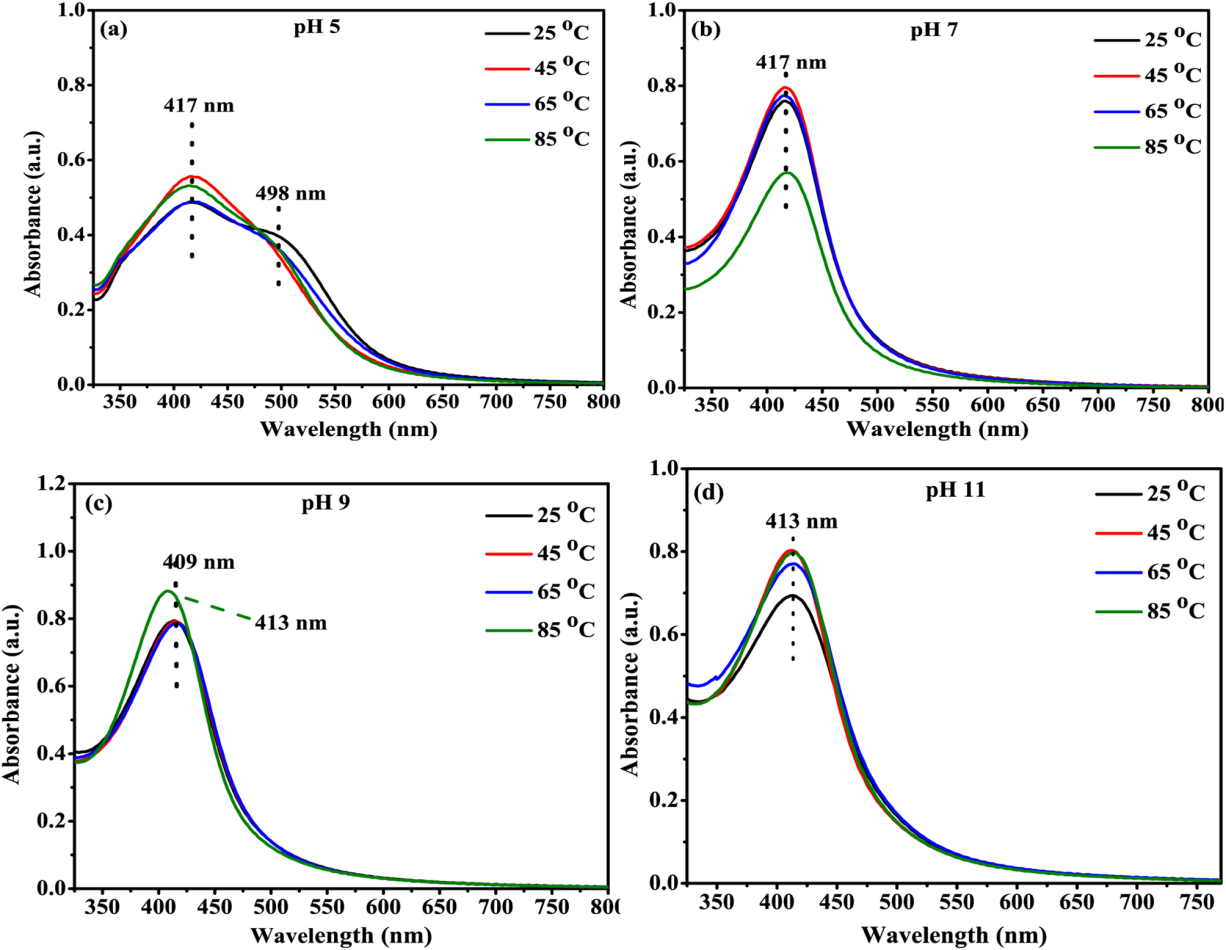
(**a**–**d**) UV–Vis absorption spectra of silver nanoparticles synthesized at different temperature for different pH conditions.

In Fig. 2a, the absorption spectra at temperature 25 °C showed an SPR band at 417 nm followed by a shoulder at 498 nm (as discussed before) implying the formation of irregularly shaped AgNPs^33^. However, with the increase in temperature from 25 to 65 °C, the shoulder diminished but doesn’t disappear completely with no shifting in the position of the SPR absorption maxima at 417 nm. Thus, it can be inferred that at pH 5 and 65 °C, the formation of predominantly spherical particles of different sizes takes place. For varying temperatures at pH 7 (Fig. 2b), a single bell-shaped peak implied the existence of spherically shaped AgNPs^23^. At pH 7 the formation of the spherical shape of the AgNPs may be due to a particular type of synchronization between the reduction, nucleation and growth process^34^. Furthermore, no shifting in the SPR peak position depicted that no significant change took place in the size range of nanoparticles with the increase in temperature. However, at pH 9 (Fig. 2c), a blue shift in the position of absorption from 413 to 409 nm was noticed with the increase in temperature from 25 to 85 °C. The small blue shift might be due to the shifting of the size range of nanoparticles toward the lower end. It can be easily believed^35^ that at a higher temperature, the reaction rate increases which favor the nucleation process, thus leading to the formation of more nuclei in comparison to the secondary reduction on the preformed nuclei^30^. Consequently, most of the reactants get utilized for the preparation of smaller nanoparticles^36,37^. In contrast, lower-temperature leads to the growth of AgNPs i.e. NPs could grow better and large at lower temperature conditions. Furthermore, at pH 11 (Fig. 2d), no shifting in the SPR band was observed with the increase in temperature from 25 to 85 °C indicating no change in the size of the AgNPs. It can thus be concluded that the AgNPs prepared under low pH (pH 5) are mostly irregular in shape and larger in size, in comparison to the AgNPs prepared under high pH (7, 9, and 11), which are more regular, spherical in shape and small in size. Such findings agree with the earlier reports^34^.

The DLS measurements (Table 1) of the prepared nanoparticles were recorded in water and implied that the average hydrodynamic diameter of prepared nanoparticles decreases with the rise in temperature from 25 to 85 °C at all tested pH during synthesis conditions. Further one can notice that there is a decrease in hydrodynamic size with the increase in pH from 5 to 9 at all corresponding temperatures which are in consonance with the UV–Vis studies.

**Table 1:** Hydrodynamic size, zeta potential, average particle size values for different pH at varying temperatures.

pH	Temperature (°C)	Hydrodynamic size (nm)	Zeta potential (mV)	TEM particle size (nm)
5	25	66.2 ± 10.6	− 15.4 ± 0.6	–
5	45	17.1 ± 4.7	− 42.9 ± 1.7	–
5	65	12.0 ± 5.6	− 33.5 ± 2.3	8.7 ± 3.9
5	85	5.2 ± 0.8	− 30.1 ± 2.5	–
7	25	17.8 ± 12.7	− 25.1 ± 3.0	16.9 ± 17.1
7	45	10.7 ± 5.0	− 30.1 ± 1.9	8.8 ± 10.4
7	65	7.5	− 32.5 ± 4.2	9.9 ± 1.7
7	85	4.9 ± 3.6	− 32.2 ± 3.9	4.3 ± 1.6
9	25	11.5 ± 11.3	− 25.5 ± 4.9	–
9	45	9.8 ± 2.0	− 28.5 ± 2.4	–
9	65	7.2 ± 0.8	− 30.8 ± 1.2	5.6 ± 2.2
9	85	6.1 ± 1.1	− 30.1 ± 2.7	–
11	25	27.0 ± 2.3	− 25.2 ± 1.3	–
11	45	13.8 ± 2.0	− 31.2 ± 1.7	–
11	65	12.4 ± 1.0	− 15.3 ± 1.9	10.4 ± 1.9
11	85	9.1 ± 0.9	− 31.4 ± 3.0	–

In our study, the average zeta potential values were also measured for the synthesized AgNPs under varied pH and temperature conditions (Table 1). The stability of a colloidal dispersion can be gauzed from zeta potential measurement as it reflected the ability of the nanoparticles to repel each other electrostatically. It measures interaction between the particles in colloidal dispersions. Moreover, the positive or negative sign value represents whether the positive or negative forces are dominant on the particle surface. So, if the particles have large negative or positive zeta potential values, then repulsive forces come into play among particles which limit the chances of agglomeration. However, if they have zeta potential values close to zero, they may undergo aggregation owing to the absence of any repulsive forces. The values for the synthesized AgNPs varied from − 15.32 ± 1.98 mV to − 42.90 ± 1.74 mV respectively. These values can be attributed to the presence of the negatively charged polyphenolic species^23^ on the surface of nanoparticles. For all tested pH, the magnitude of the zeta potential of particles prepared at the temperature at 85 °C was found to be higher than that of prepared at 25 °C indicating the increased stability of the AgNPs prepared at the higher temperature.

**Figure 3:**
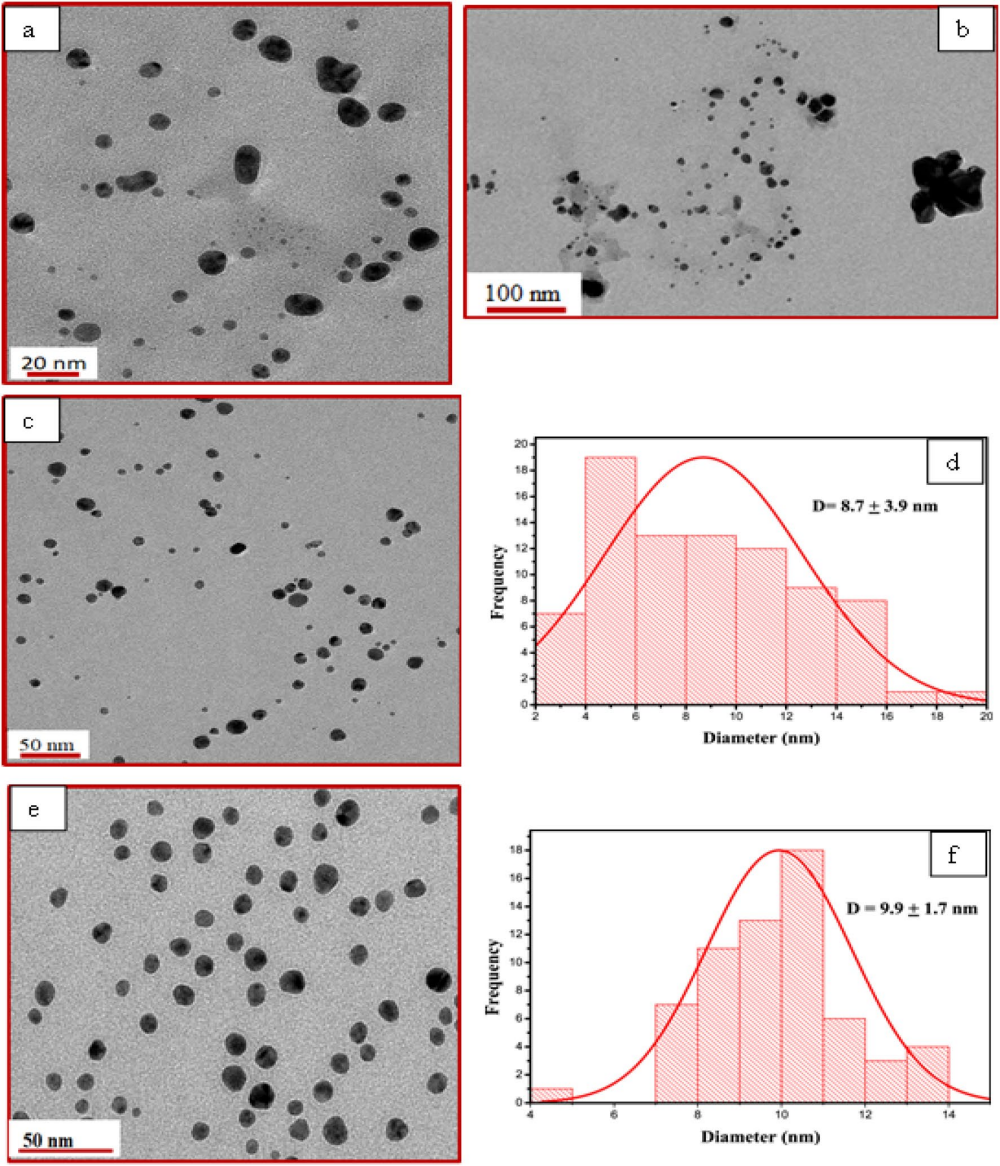
TEM images and histograms of the AgNPs prepared at pH 5 and 25 °C (**a**, **b**), pH 5 and 65 °C (**c**, **d**), and pH 7 and 65 °C (**e**, **f**).

**Figure 4:**
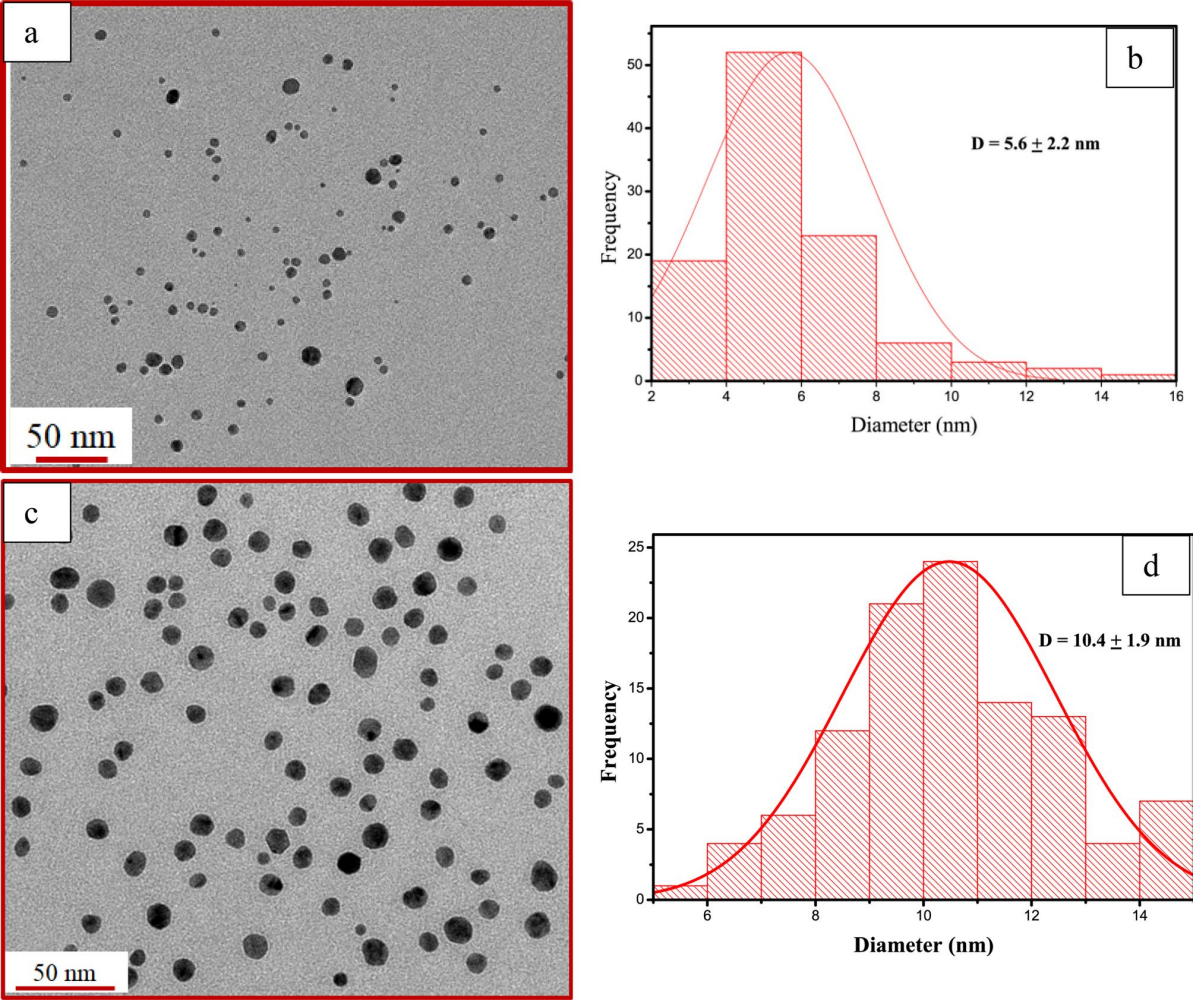
TEM images and corresponding histograms of the AgNPs prepared at pH 9 and 65 °C (**a**, **b**), and pH 11 and 65 °C (**c**, **d**).

TEM micrographs of the AgNPs prepared at pH 5 and 25 °C (Fig. 3a,b) represent irregularly shaped particles. However, as the synthesis temperature increased from 25 to 65 °C at the same pH, spherical particles in the range of 2 nm to 20 nm were formed as shown in (Fig. 3c,d). The average particle diameter was found to be 8.7 nm with the highest standard deviation (± 3.9). These findings align with UV–visible spectra where a broad absorption was observed at these reaction conditions indicating wide particle size distribution. With the increase in temperature during synthesis, the reaction rate increases, and the formation of spherical nanoparticles takes place. A similar trend was observed with the increase in temperature at pH 7 (Fig. S1). As the temperature was increased from 25 to 85 °C, the size of the nanoparticles was found to decrease which is in agreement with the DLS analyses. Therefore, considering these findings and DLS studies, micrographs of all samples prepared at 65 °C for different pH 7, 9, and 11 were recorded. The image (Fig. 3e) of the sample prepared at pH 7 and temperature 65 °C depicted the formation of highly dispersed and spherical particles with an average diameter of 9.9 nm with the least standard deviation (± 1.7) (Fig. 3f). With a further increase in pH from 7 to 9, the formation of dispersed particles (Fig. 4a) with an average size of 5.6 nm (Fig. 4b) was observed. This decrease in the average particle size can be ascribed to the efficient reduction of the silver ions facilitated by the presence of hydroxide ions in the solution as discussed earlier. However further increase in pH to 11 doesn’t reduce the size, rather, a small increase in the average size of 10.4 nm as can be seen in (Fig. 4c,d). This means an increase in pH up to 9 during synthesis can help decrease the size of particles. These findings agreed with the DLS studies. However, the hydrodynamic size determined from DLS was observed to be higher than the average size obtained from TEM studies which may be due to the existence of layers of hydrated phytochemicals on the surface of nanoparticles.

Furthermore, the EDX spectra at pH 11 and 65 °C (Fig. S2a) showcased a peak at 3 keV which can be assigned to elemental nanocrystalline silver^31^. Furthermore, the O peak arises from the polyhydroxy groups in the catechins that reduce the Ag ions to metallic AgNPs and Na peak from the NaOH used for maintaining the pH while the Cu peak appeared due to the Cu grid used in measurement^38^. SAED pattern (Fig. S2b) also supported the existence of the Ag (111) plane of crystalline Ag NPs.

Powder XRD pattern (Fig. S3) of AgNPs prepared at pH 11 and 65 °C was recorded to confirm their identification of the nanoscale crystallographic structure. It depicted five reflections at 38.4°, 44.2°, 64.5°, 77.7°, 81.6° corresponding to the (111), (200), (220), (311), and (222) planes respectively which can be accorded with JCPDS-04-083 of silver. These observations verified the fact that silver nanoparticles have a face-centered cubic structure^39^. Besides, the crystallite size of 12.5 nm was determined by prepared nanoparticles using the Debye–Scherrer formula by considering reflection corresponding to (111) plane. These reflections justified the nanoscale and crystalline nature of the prepared AgNPs. However, crystallite size can be equal to particle size predicted by TEM if the prepared material is in the form of single-crystalline and under no circumstance, it can be higher than the particle size. In TEM studies of this material, particles of 5 to 15 nm were observed.

FTIR spectra (Fig. S4) of green tea extract and thoroughly washed AgNPs (prepared pH 9 and 65 °C) were recorded to elucidate the interactions between the extract and the synthesized AgNPs. Both spectra consisted of a strong band around 3250 cm^–1^ corresponding to the stretching vibrations of –OH groups of polyphenols (e.g. catechins) and bands at 2921 cm^–1^ and 1033 cm^–1^ can be ascribed to stretching vibrations of *sp*^2^ hybridized C–H bonds of the hydrocarbons^40^. A weak band at 1606 cm^–1^ in the tea extract suggests the presence of C=O stretching vibrations of acidic functional groups present in the natural compounds of green tea^40^. The transmittance at 1224 cm^–1^ can be ascribed to the stretching vibrations of C–O bonds while a small band at 819 cm^–1^ confirmed the existence of aromatic substituted rings. Thus, the similarities between both the FTIR spectra, with slight variation in transmittance illustrated that natural constituents of the extract are present along with nanoparticles. The presence of such constituents was confirmed through the HR-TEM image (Fig. S5a) where the difference in the electron density signified the occurrence of natural constituents on the surface of the AgNPs. It was found to agree with the EDX findings (Fig. S5b) that verified the existence of carbon and oxygen-containing functional groups in the prepared material.

**Scheme 1:**
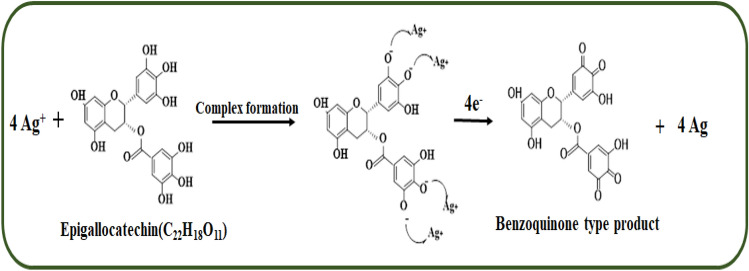
Proposed mechanism of reduction of metal ions by epigallocatechin (a commonly found polyphenol in green tea extract).

Moreover, as seen in TEM images that these nanoparticles are present in a well-dispersed form, thus it can be believed that these constituents were decorated on the surface of particles and were acting as stabilizing agents^16,41^. In a recent report^42^, the preparation of silver nanoparticles has been proposed using quercetin, a polyphenolic compound as a reducing agent. Though, the green tea contains a major proportion of polyphenolics ~ 30% dry weight along with fibre (26%), protein (15%), carbohydrate (7%), lipid (7%), minerals (5%), amino acids (4%), and pigments (2%) however, only polyphenolic compounds viz., catechin, gallocatechin, epicatechin, epigallocatechin, etc. can act as reducing agent thus reducing silver ions to AgNPs. Keeping in view these findings, the following mechanism (Scheme 1) is being proposed for the synthesis of AgNPs using epigallocatechin.

### Antibacterial activity

Antibacterial activities of prepared AgNPs at 65 °C and pH range (5–11) were investigated towards gram-negative (*E. coli*) and gram-positive (*S. aureus*) strains of bacteria using agar well diffusion methods. As can be seen in Fig. S6, the zone of inhibition (ZOI) was found to be higher in both the strains when particles prepared at pH 9 were employed for the antibacterial activity. Furthermore, ZOI for *E. coli* and *S. aureus* have been displayed in Table S1 and found to be 13 ± 2.5 mm and 8.0 ± 2.5 mm respectively. Different ZOI for both the strains can be justified based on their different structural compositions of cell walls. It is expected that upon interaction with bacteria, nanoparticles bind to the cell membrane and change its physical and chemical properties which in turn malfunctioned the normal physiological process such as respiration and permeability, etc. of cells^43^. Further, these nanoparticles can also induce the formation of various reactive nitrogen and oxygen species which generate oxidative stress on DNA, and other important cell constituents, and disrupt the overall functioning of bacterial cells. Similar findings were reported elsewhere^44,45^.

### Human cervix epitheloid carcinoma cell proliferation and cytotoxicity

**Figure 5:**
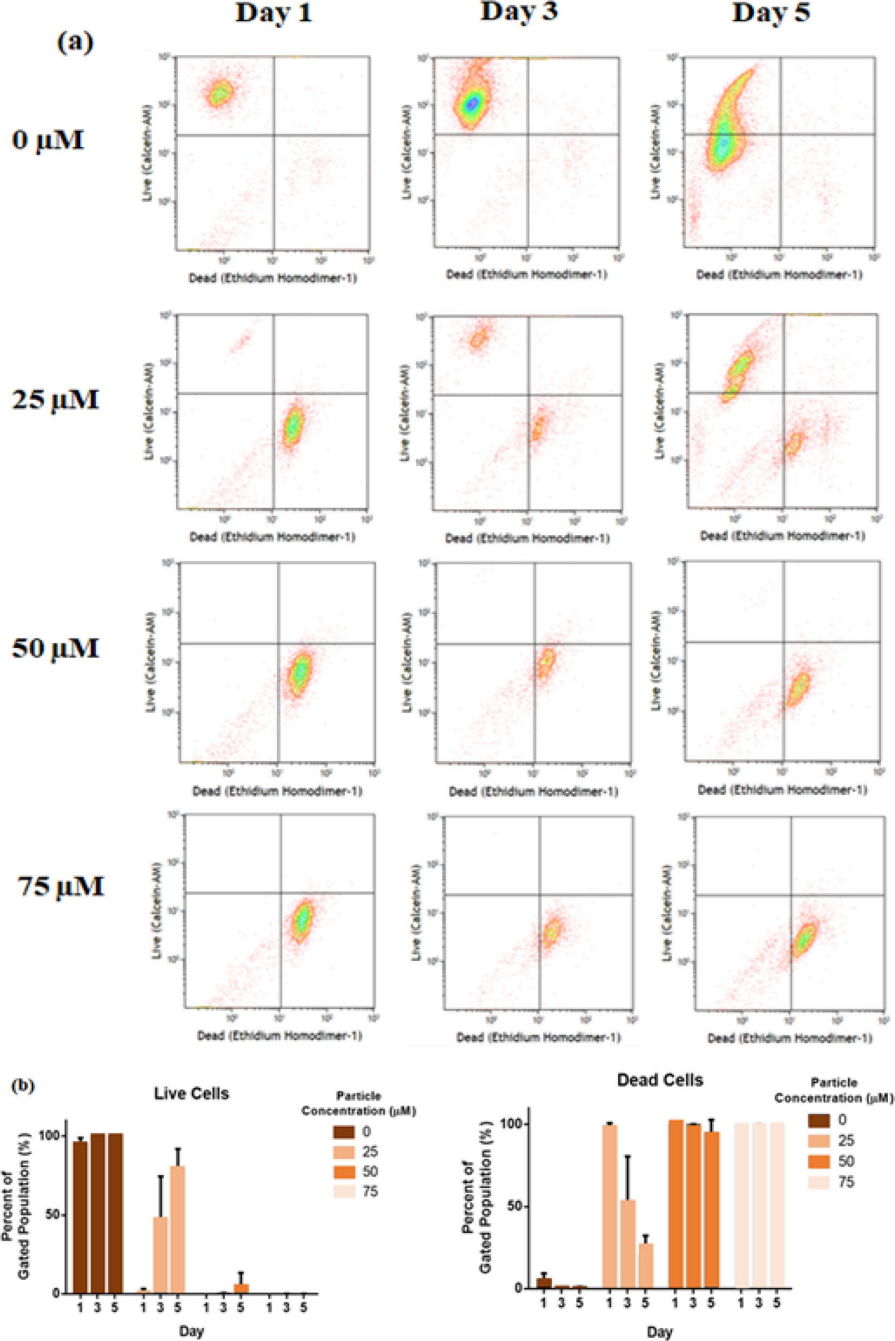
(**a**) Live/dead staining of human cervix epitheloid carcinoma cells after treatment with AgNPs (prepared at pH 9 and 65 °C), (**b**) percentage gated population versus days pH and temperature conditions.

The cytotoxicity studies (Fig. S7) of AgNPs synthesized at pH 9 and 65 °C were carried out on HeLa cells. The normal proliferative response of HeLa cells was evaluated by measuring cell count over 5 days without any AgNPs added. The addition of 25 µM AgNPs significantly decreased HeLa cell proliferation, while the addition of 50 µM and 75 µM inhibited cellular proliferation altogether. The effect in these higher concentrations appeared to be cytotoxic, as the number of cells after 1 day of incubation was significantly lower than the number of cells that were seeded in the plate. These results indicated that AgNPs hindered human cervical cancer cell growth in a dose-dependent manner. Furthermore, our findings were in agreement with other reports^46–48^ validating the cytotoxicity of AgNPs. To determine whether this change in proliferation was inhibitory or cytotoxic, FC using the LIVE/DEAD stain was performed as shown in Fig. 5a and b. Cellular growth with no addition of AgNPs was observed over the 5 days, with most of the cells being positively stained as live.

The addition of 25 µM AgNPs showed a high proportion of cells stained positively as dead, but the live population grew over the 5 days. This trend was similar to what was seen in the proliferation assay with Alamar Blue. This indicated there was a small cytotoxic effect at this concentration, though some cells survived and continued proliferating. In the high concentrations of AgNPs (50 µM and 75 µM), all cells stained positively as dead for all time points, indicating a purely cytotoxic effect. While the AgNPs appeared to have a cytotoxic effect on this cancer cell line, further evaluation with non-cancer cell lines is required to determine if this cytotoxic effect is cancer cell-specific.

## Conclusion

The synthesis of AgNPs was attempted by greener pathways using a green tea extract (Legends of China). In this work, the changes in the morphology and size distribution of AgNPs were studied owing to the changes in pH and temperature during reaction conditions. It can be inferred that a reaction temperature of 65 °C and pH 9 is optimum for the preparation of monodispersed small-sized spherical nanoparticles. All the characterization studies suggested that polyphenolic compounds of green tea acted as both reducing as well as stabilizing agents which afforded the uniform nucleation and growth of silver nanoparticles in a highly disperse and stabilized state. The prepared silver nanoparticles showcased persuasive antibacterial activity against both gram-positive and negative bacterial strains. Finally, both the proliferation and flow cytometry analysis of HeLa cells treated with AgNPs demonstrated a dose-dependent cytotoxic effect on the cells.

## Supplementary Information

Supplementary Information.
