# Supplementary material for: Exceptional antibacterial and cytotoxic potency of monodisperse greener AgNPs prepared under optimized pH and temperature
Source: Sci Rep. 2021 Feb 3;11:2866. doi: 10.1038/s41598-021-82555-z (PMC7858571; doi:10.1038/s41598-021-82555-z)
Supplement: Supplementary file 1 — Supplementary Information. [file 41598_2021_82555_MOESM1_ESM.docx]

**Supporting Information**

Exceptional Antibacterial and Cytotoxic potency of Monodisperse Greener AgNPs prepared under optimized pH and temperature

Muhammad Riaz^1,2^, Vishal Mutreja^1,3^_,_ Shweta Sareen^4^, Bashir Ahmad^2^, Muhammad Faheem^2^, Nafeesa Zahid^5^, Ghassan Jabbour^1^, and Jeongwon Park^1,6^*

^1^School of Electrical Engineering and Computer Science, University of Ottawa, Ottawa, ON, K1N 6N5, Canada

^2^Department of Biological Sciences, International Islamic University Islamabad, 44000, Pakistan

# ^3^Division Chemistry, University Institute of Sciences, Chandigarh University, Gharuan, Mohali, Punjab, India

# ^4^Department of Chemistry, Panjab University, Sector-14, Chandigarh, India

# ^5^Department of Botany, Mirpur University of Science and Technology (MUST), Mirpur Azad Kashmir 10250, Pakistan

# ^6^Department of Electrical and Biomedical Engineering, University of Nevada, Reno, NV 89557, USA

# *Email: jepark@unr.edu

**Contents**

Determination of concentration of silver nanoparticles.

**Figure S1.** TEM images and corresponding histograms of the AgNPs prepared at pH 7 and temperatures, 25 ^o^C **(a-b),** 45 ^o^C **(c-d)** and 85 ^o^C **(e-f).**

**Figure S2(a)** EDX spectra and **(b)** SAED pattern of the prepared AgNPs using green tea extract prepared at pH 11 and 65 ^o^C.

**Figure S3.** Powder XRD pattern of prepared silver nanoparticles pH 11 and 65 ^o^C.

**Figure S4.** FTIR spectra of the extract of green tea and prepared nanoparticles prepared at pH 9 and 65 ^o^C.

**Figure S5(a)** HR-TEM **and (b)** EDX spectra of the AgNPs prepared using green tea extract at pH 9 and 65 ^o^C.

**Figure S6.** Zone of inhibition of E. coli **(a-b)** and S. aureus **(c-d).**

**Figure S7.** Human Cervix Epitheloid Carcinoma Cell Proliferation after Treatment with AgNPs prepared at pH 9 and 65 ^o^C.

**Table S1.** Antimicrobial properties (ZOI) of AgNPs tested using different bacterial strains.

**Determination of concentration of silver nanoparticles**

The concentration of silver nanoparticles was determined as reported elsewhere ^1,2^. The calculation was done as follows:

1. To determine the number of silver nanoparticles

Initially, the number of silver atoms per nanoparticle was determined using the formula (assuming 100 % consumption of Ag ions to AgNPs).

$N= \frac{\pi\rho D^{3}}{6M}.N_{A}$

*Where N is the number of atoms per nanoparticle, π* = 3.14, ρ is the density of face centered cubic (fcc) silver (10.5 g/cm^3^), D is the average diameter of nanoparticles, M is the atomic mass of silver ( 107.8 g), N_A_ is the number of atoms per mole (Avogadro’s number) (6.023 × 10^23^).

1. To determine the amount of nanoparticles formed when 100 mL of 1 mM AgNO_3_ is reduced by the green extract

Moles of AgNO_3_ = 0.1 L × 1 × 10^-3^ = 10^-4^ moles

No. of silver atoms = 10^-4^ × 6.023 × 10^23^ = 6.023 × 10^19^

No. of Nanoparticles (N_NP_) = $\frac{No. of Ag atoms}{N}$

Since 6.023 × 10^23^ of NPs are present in 1 mol/L

Concentration of nanoparticles (C_NP_) = $\frac{N_{NP}}{6.023 \times{10}^{23}}$


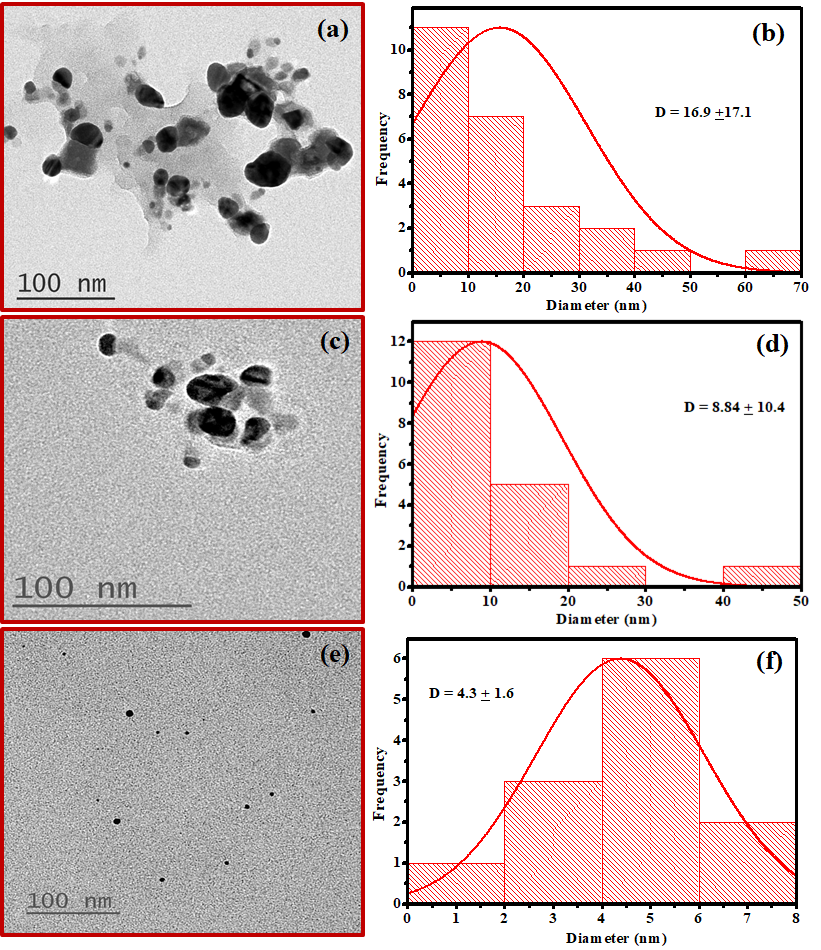


Figure S1. TEM images and corresponding histograms of the AgNPs prepared at pH 7 and temperatures, 25 ^o^C (a-b), 45 ^o^C (c-d) and 85 ^o^C (e-f).


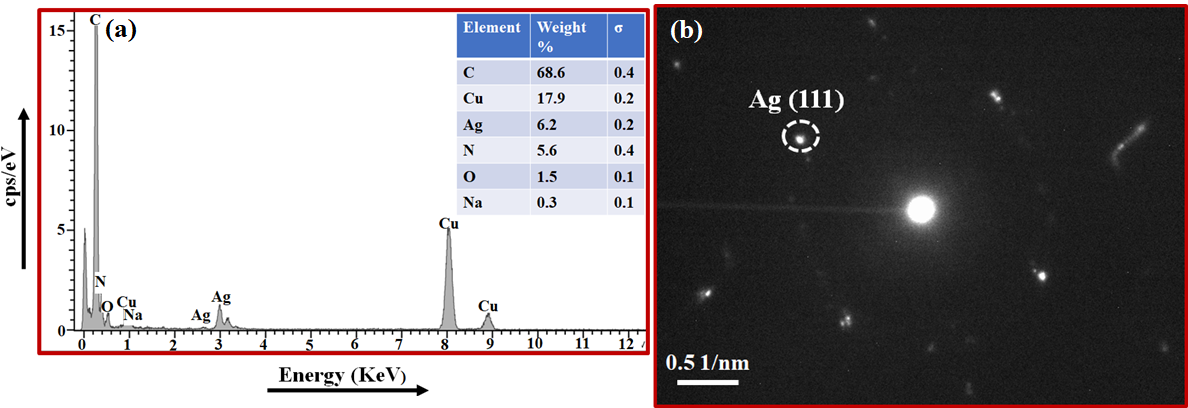


Figure S2(a) EDX spectra and (b) SAED pattern of the prepared AgNPs using green tea extract prepared at pH 11 and 65 ^o^C.


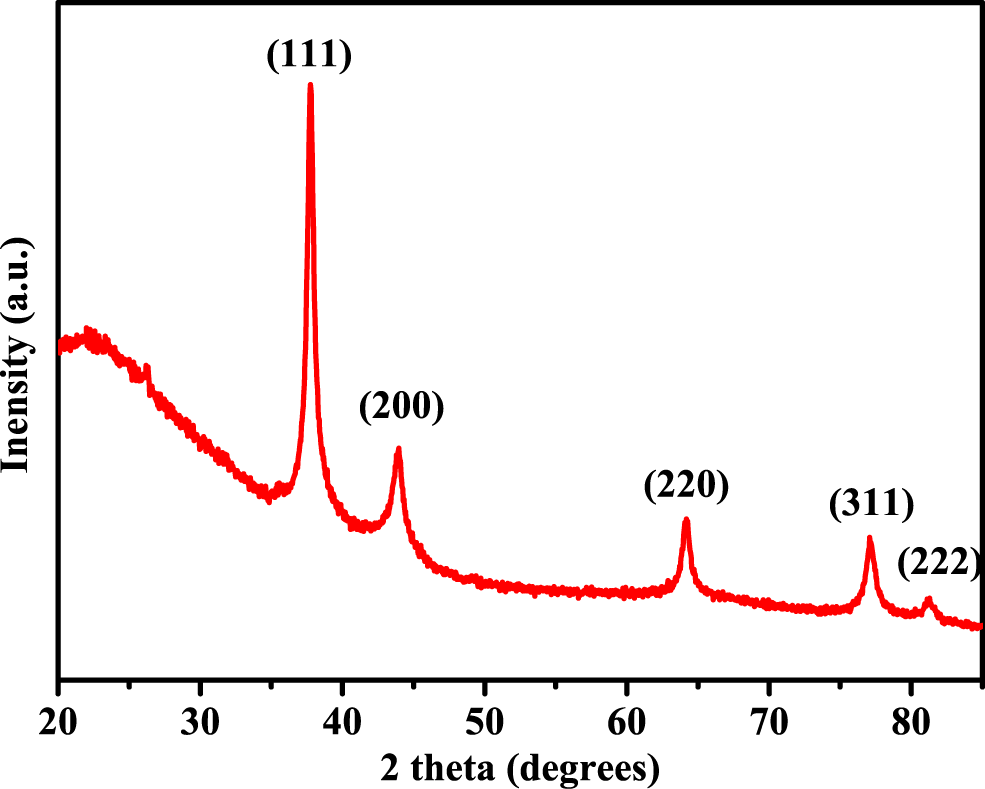


**Figure S3.** Powder XRD pattern of prepared silver nanoparticles pH 11 and 65 ^o^C.

**
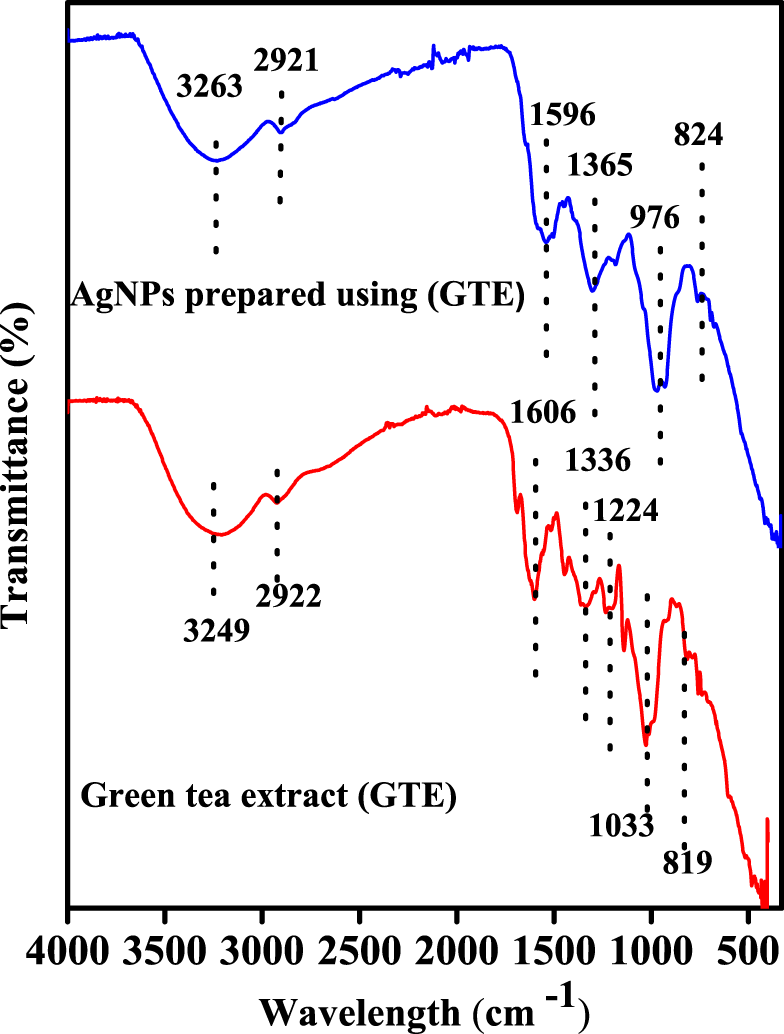
**

**Figure S4.** FTIR spectra of the extract of green tea and prepared nanoparticles prepared at pH 9 and 65 ^o^C.


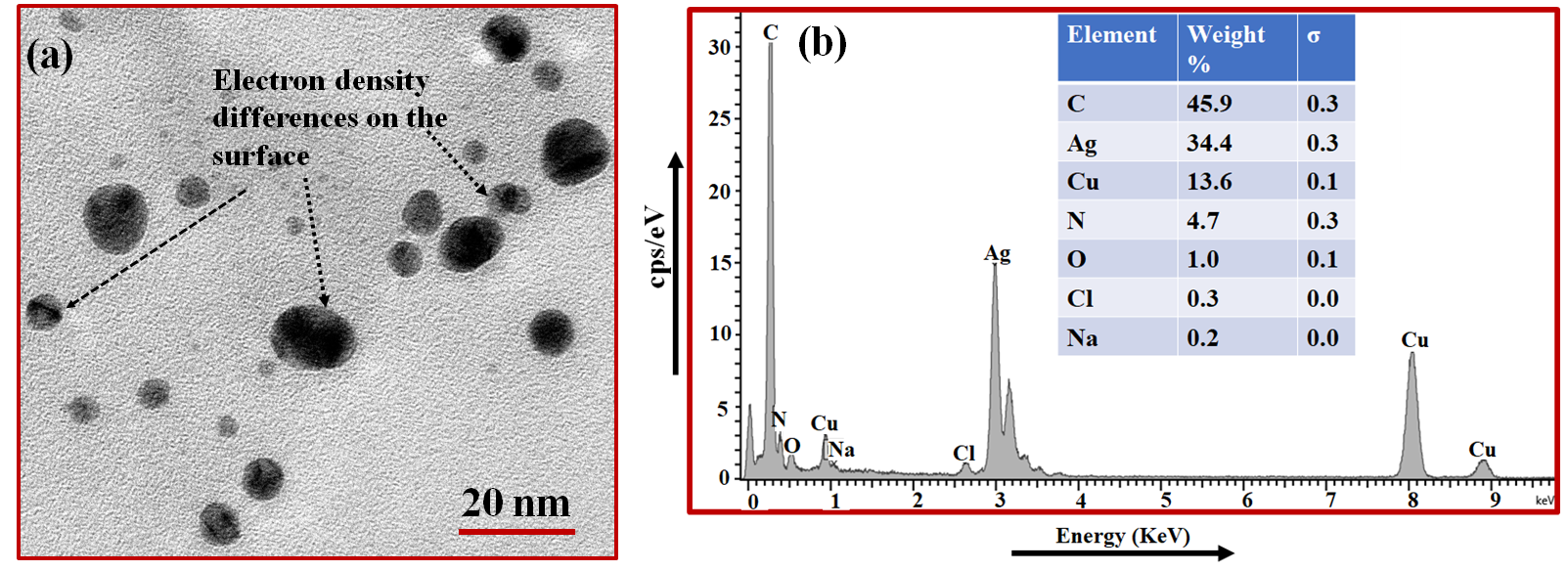
Figure S5(a). HR-TEM and (b) EDX spectra of the AgNPs prepared using green tea extract at pH 9 and 65 ^o^C.

**Table S1.** Antimicrobial properties (ZOI) of AgNPs tested using different bacterial strains

| **Code** | **Type of materials/conc.** | **ZOI in mm (*E. coli*)** | **ZOI in mm (*S. aureus*)** |
| --- | --- | --- | --- |
| 1 | pH5/65^o^C /20µM | 10±2.0 | 3.5±1.0 |
| 2 | pH7/65^o^C /20µM | 12±1.5 | 2.3±1.5 |
| 3 | pH11/65^o^C /20µM | 11±2.0 | 4.0±2.0 |
| 4 | pH9/65^o^C /20µM | 13±2.5 | 8.0±2.5 |
| 5 | pH9/65^o^C /10µM | 9.0±1.0 | 5.0±1.0 |
| 10 | Positive Control/5µM | 10±1.5 | 7.9±2.5 |
| c | Negative Control | 00±00 | 00±00 |

Figure S6. Zone of inhibition of silver nanoparticles against E. coli (a-b) and S. aureus (c-d).


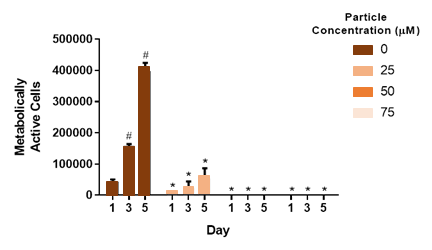


**Figure S7.** Human Cervix Epitheloid Carcinoma Cell Proliferation after Treatment with AgNPs prepared at pH 9 and 65 ^o^C.

**References**

1. Kalishwaralal, K., BarathManiKanth, S., Pandian, S. R. K., Deepak, V. & Gurunathan, S. Silver nanoparticles impede the biofilm formation by Pseudomonas aeruginosa and Staphylococcus epidermidis. *Colloids Surfaces B Biointerfaces* **79**, 340–344 (2010).

2. Lewis, D. J., Day, T. M., MacPherson, J. V & Pikramenou, Z. Luminescent nanobeads: attachment of surface reactive Eu(III) complexes to gold nanoparticles. Experimental - Calculations concentrations Au-NPs. *Chem. Commun. (Camb).* **168**, (2006).
